# Supplementary material for: Truncated Analogues of a G-Quadruplex-Forming Aptamer Targeting Mutant Huntingtin: Shorter Is Better!
Source: Int J Mol Sci. 2022 Oct 17;23(20):12412. doi: 10.3390/ijms232012412 (PMC9604342; doi:10.3390/ijms232012412)
Supplement: Supplementary file 1 [file ijms-23-12412-s001.zip › ijms-1943581-supplementary.pdf]

Supplementary Materials

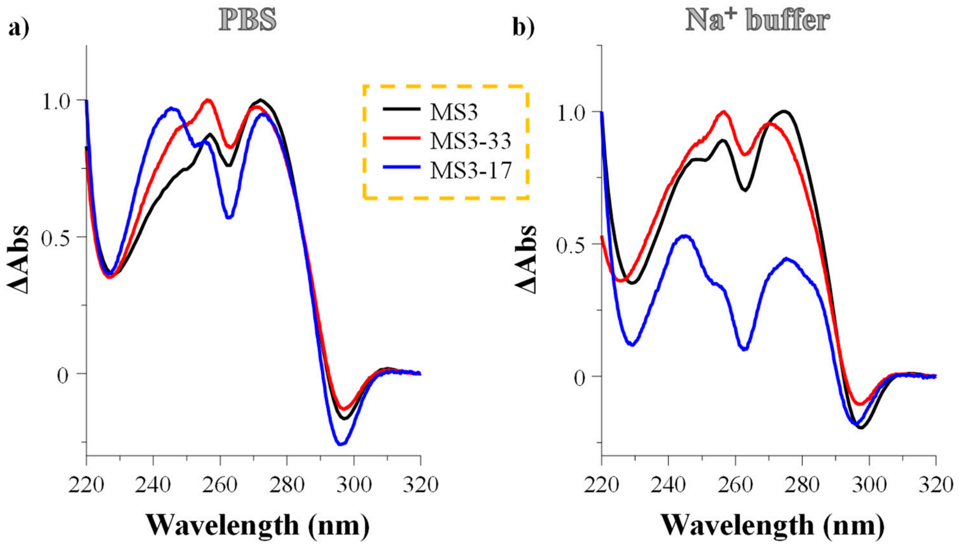

Figure S1. Representative normalized TDS profiles of MS3-33 and MS3-17 analogues (red and blue line, respectively) at 2 μM concentration in PBS (**a**) and Na^+ buffer (**b**) solutions, in comparison with their MS3 precursor (black line). TDS profiles are obtained for each oligonucleotide by subtracting the UV spectrum registered at 15 °C from the one recorded at 100 °C.

Table S1. TDS factors, i.e. ratios between differences in absorbance values at different wavelengths as calculated for the MS3 analogues in the selected saline conditions from normalized TDS spectra, according to literature protocols [1].

	**TDS factors**					
	**PBS**			**Na^+ buffer**		
	$\Delta A_{240}/\Delta A_{295}$	$\Delta A_{255}/\Delta A_{295}$	$\Delta A_{275}/\Delta A_{295}$	$\Delta A_{240}/\Delta A_{295}$	$\Delta A_{255}/\Delta A_{295}$	$\Delta A_{275}/\Delta A_{295}$
**MS3**	**4.5**	**6.3**	**7.2**	**4.3**	**5.8**	**6.6**
**MS3-33**	**7.0**	**9.5**	**9.0**	**8.8**	**12.1**	**11.1**
**MS3-17**	**3.6**	**3.4**	**3.7**	**2.6**	**2.0**	**2.5**

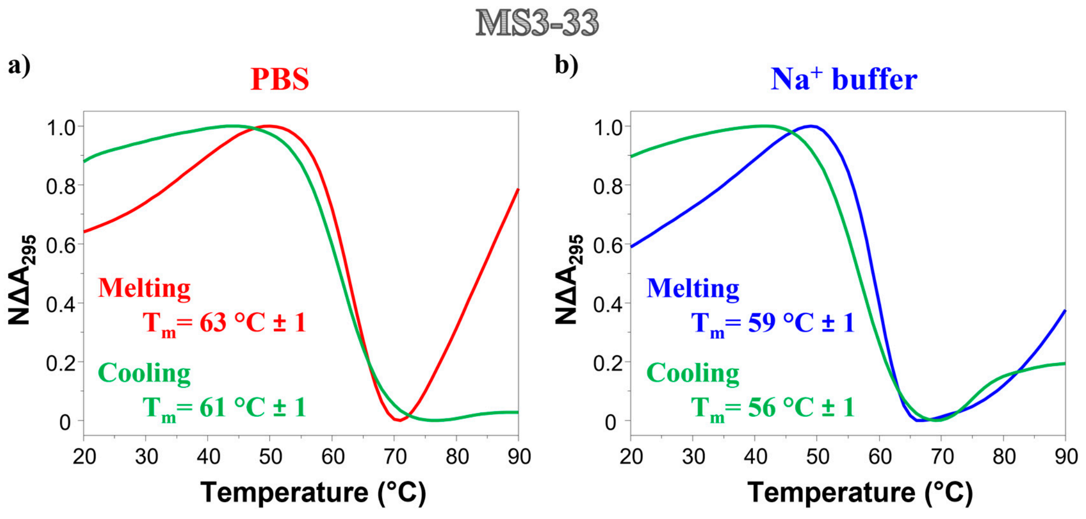

Figure S2. Representative normalized UV-melting and UV-cooling profiles of MS3-33 at 2 μM concentration in PBS (**a**) and Na^+-buffer (**b**) solutions. The UV-monitored thermal curves were recorded following the signal at 295 nm in both saline conditions, with a temperature scan rate of 1 °C min^{-1}.

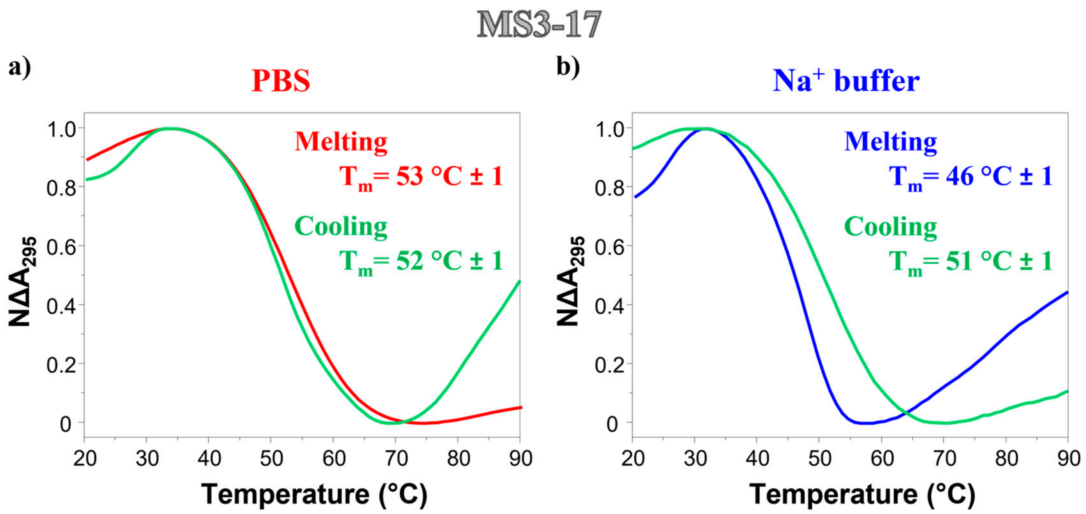

Figure S3. Representative normalized UV-melting and UV-cooling profiles of MS3-17 at 2 μM concentration in PBS (**a**) and Na$^+$-buffer (**b**) solutions. The UV-monitored thermal curves were recorded following the signal at 295 nm in both saline conditions, with a temperature scan rate of 1 °C min^{-1}.

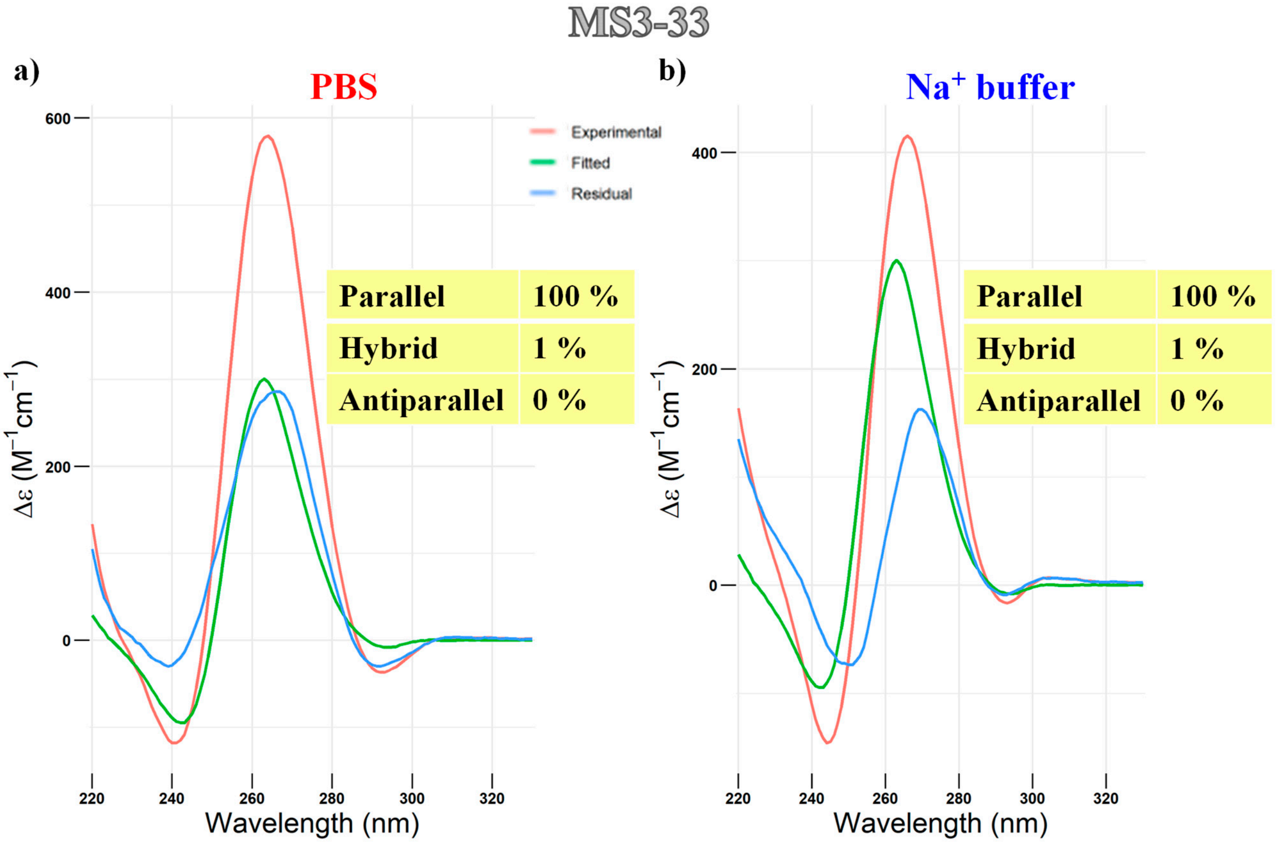

Figure S4. Prediction of the relative abundance of the G4 topologies adopted by MS3-33, obtained by singular value decomposition (SVD) analysis of the CD spectra recorded in PBS (**a**) and Na$^+$ buffer (**b**) solutions, performed by exploiting the software developed by del Villar-Guerra et al. [2]. Deviations from 100% (± 1%) are due to significant digits approximation of the values originally obtained by the simulations.

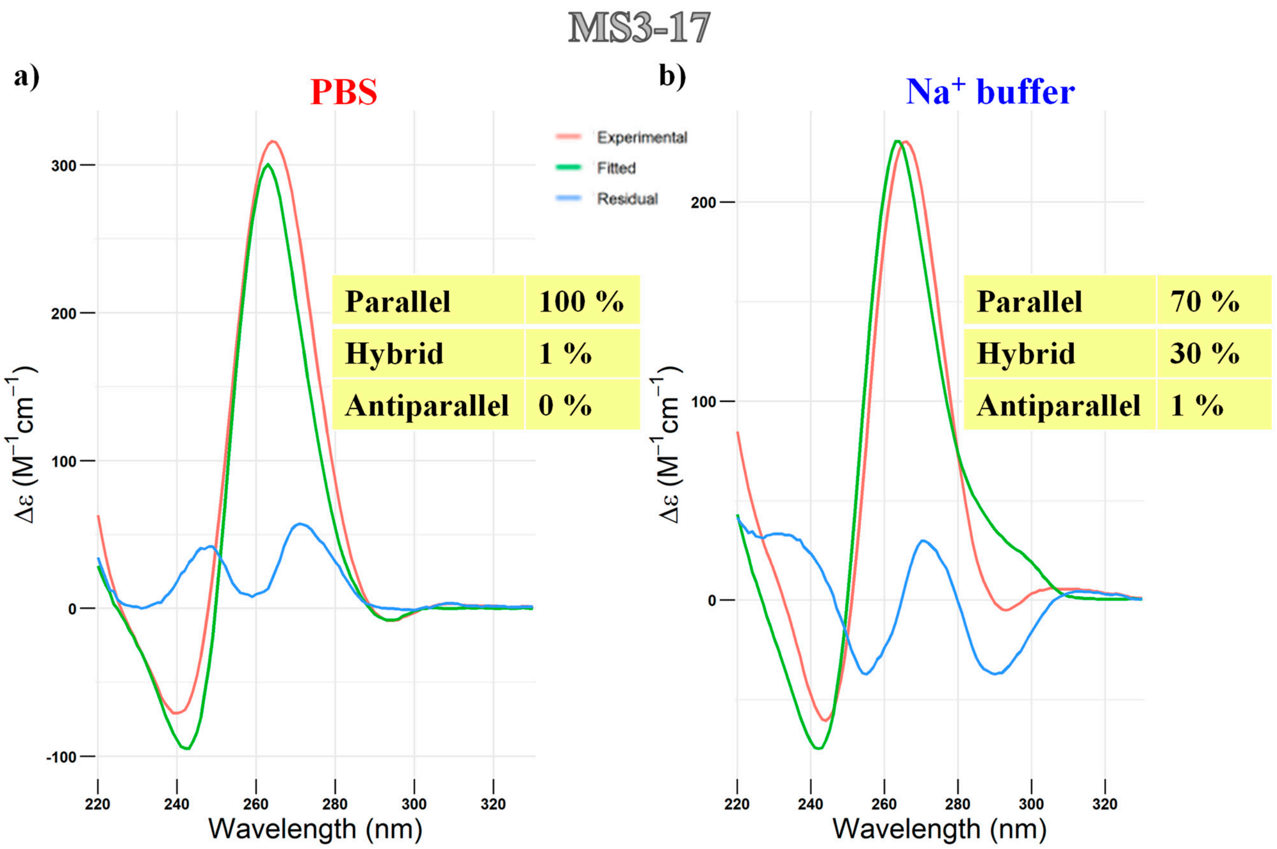

Figure S5. Prediction of the relative abundance of the G4 topologies adopted by MS3-17, obtained by singular value decomposition (SVD) analysis of the CD spectra recorded in PBS (**a**) and Na^+ buffer (**b**) solutions, performed by exploiting the software developed by del Villar-Guerra et al. [2]. Deviations from 100% (± 1%) are due to significant digits approximation of the values originally obtained by the simulations.

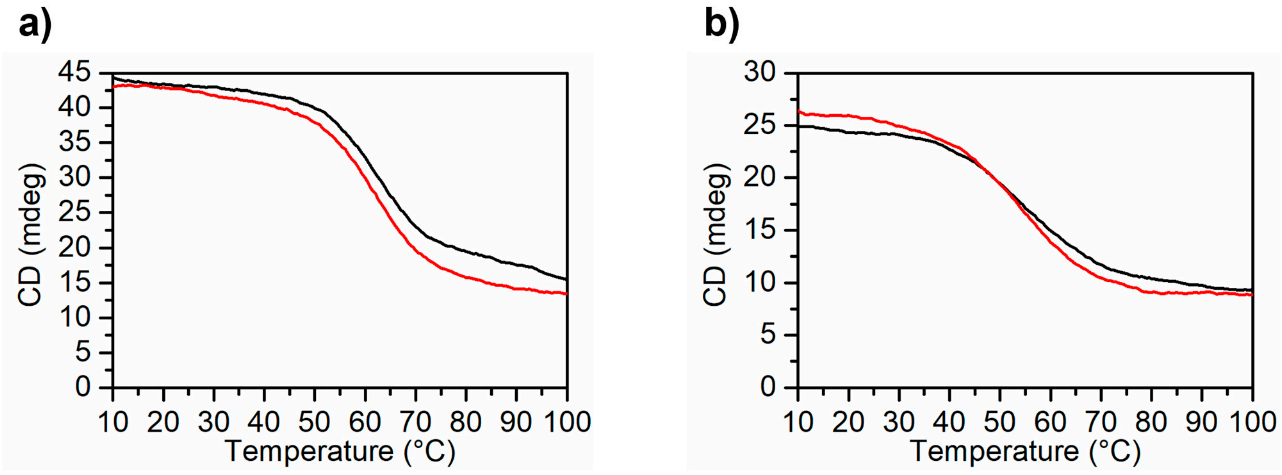

Figure S6. CD-melting experiments of MS3 analogues registered at the scan rate of 0.5 °C min^{-1}: CD-melting (black line) and cooling (red line) profiles of MS3-33 (**a**) and MS3-17 (**b**) in PBS buffer.

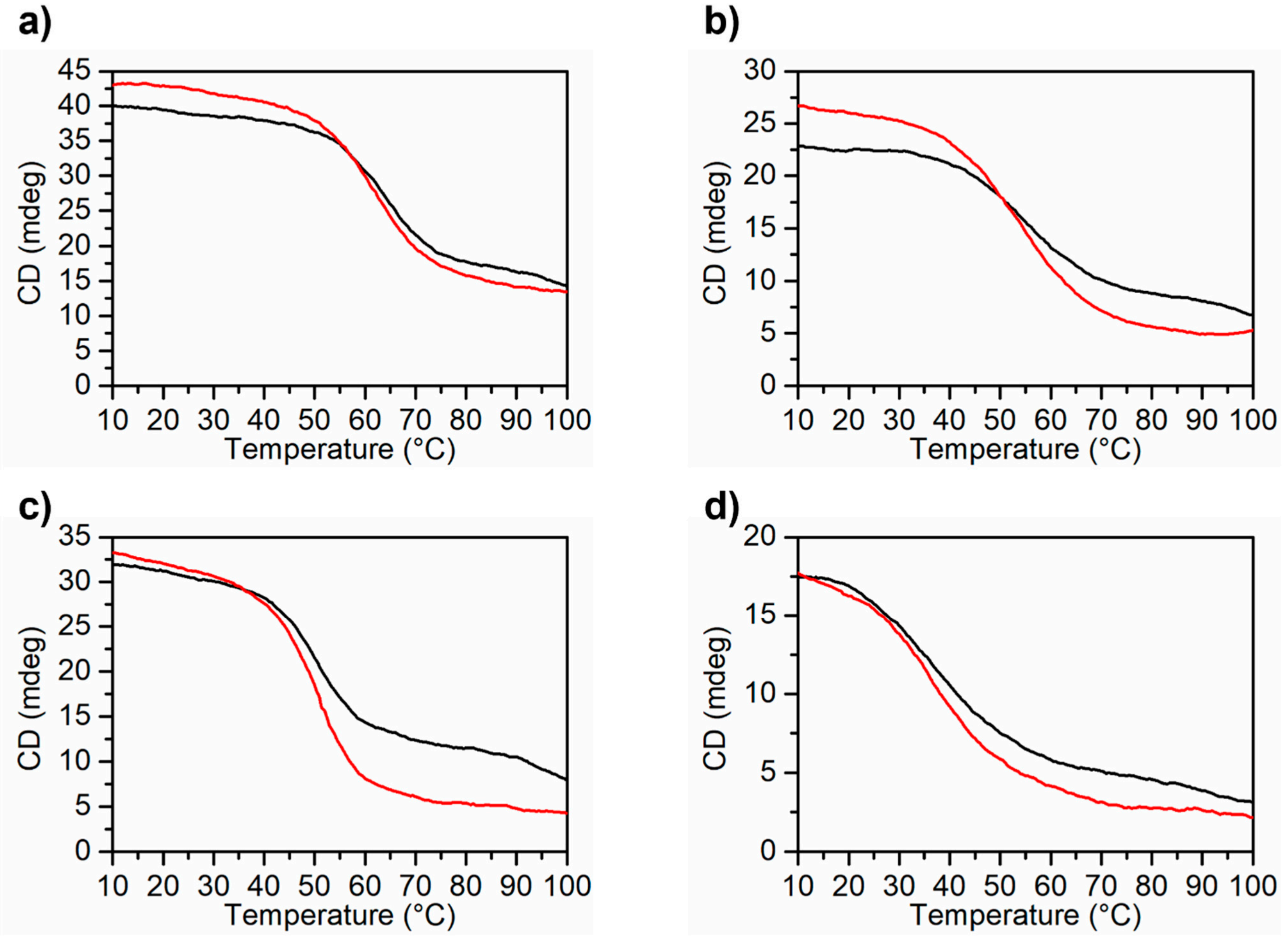

Figure S7. CD-melting experiments of MS3 analogues registered at the scan rate of 1 °C min^{-1}: CD-melting (black line) and cooling (red line) profiles of MS3-33 (**a**) and MS3-17 (**b**) in PBS buffer; CD-melting experiments of MS3-33 (**c**) and MS3-17 (**d**) in the Na^+ buffer.

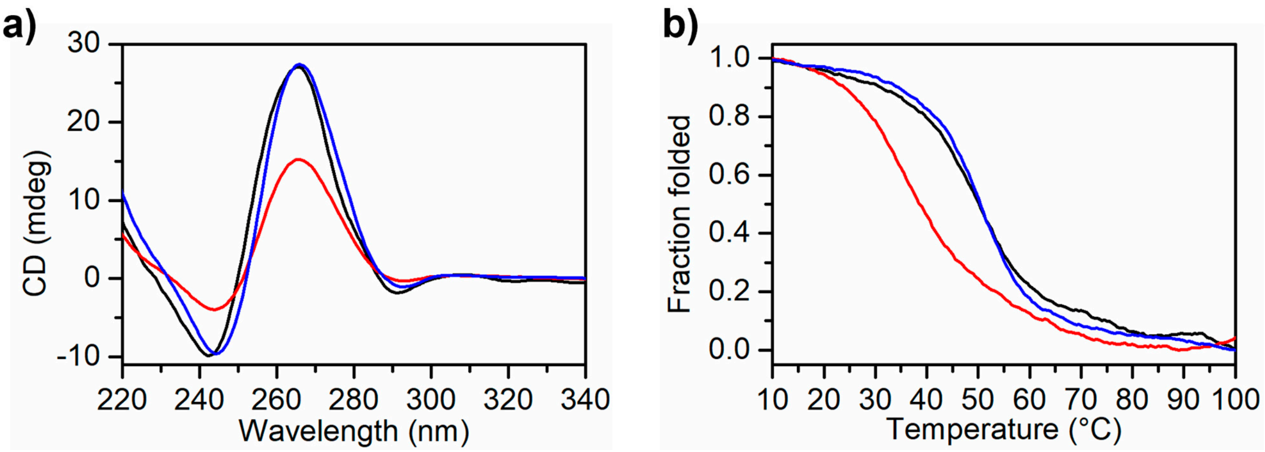

Figure S8. CD analysis of MS3 and MS3 analogues: (**a**) overlapped CD spectra of MS3 (black line), MS3-33 (blue line) and MS3-17 (red line) in the Na^+ buffer (10 mM NaH_2PO_4/Na_2HPO_4, 90 mM NaCl solution, pH = 7.0). (**b**) overlapped CD-melting curves of MS3 (black line), MS3-33 (blue line) and MS3-17 (red line) registered at the scan rate of 0.5 °C min^{-1} in the Na^+ buffer (10 mM NaH_2PO_4/Na_2HPO_4, 90 mM NaCl solution, pH = 7.0).

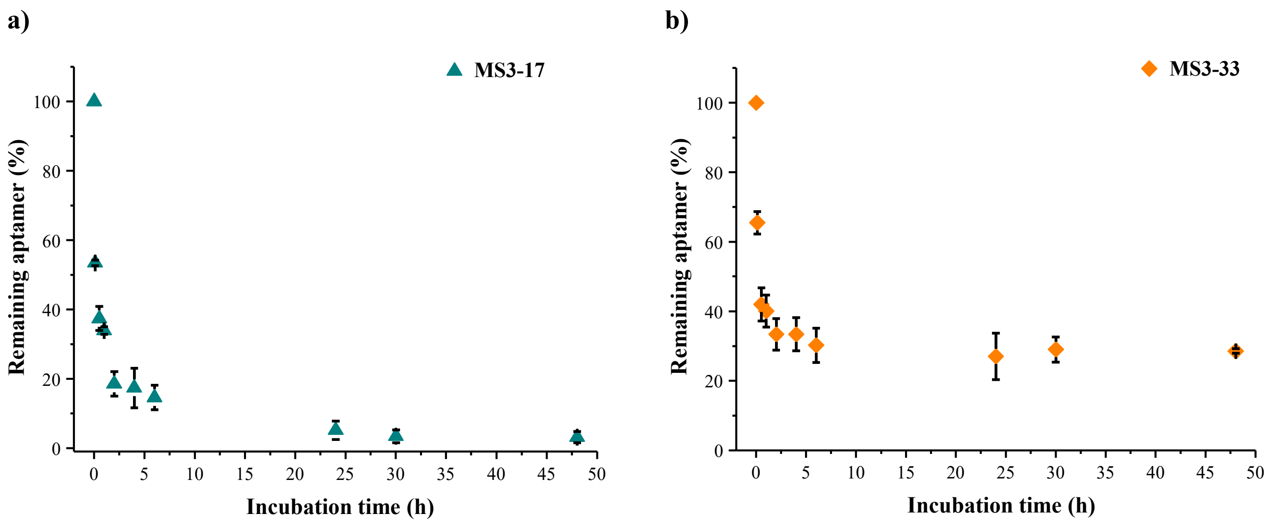

Figure S9. Time-dependent degradation of MS3-17 (**a**) and MS3-33 (**b**) analogues incubated in 80% FBS as monitored by denaturing 20% PAGE up to 48 h. The intensity of each oligonucleotide band corresponding to the intact oligonucleotide on the gel is expressed as percentage of the remaining intact aptamer with respect to that of the untreated oligonucleotide for all the analyzed time points. Data are reported as mean values ± SD (error bars) for multiple determinations.

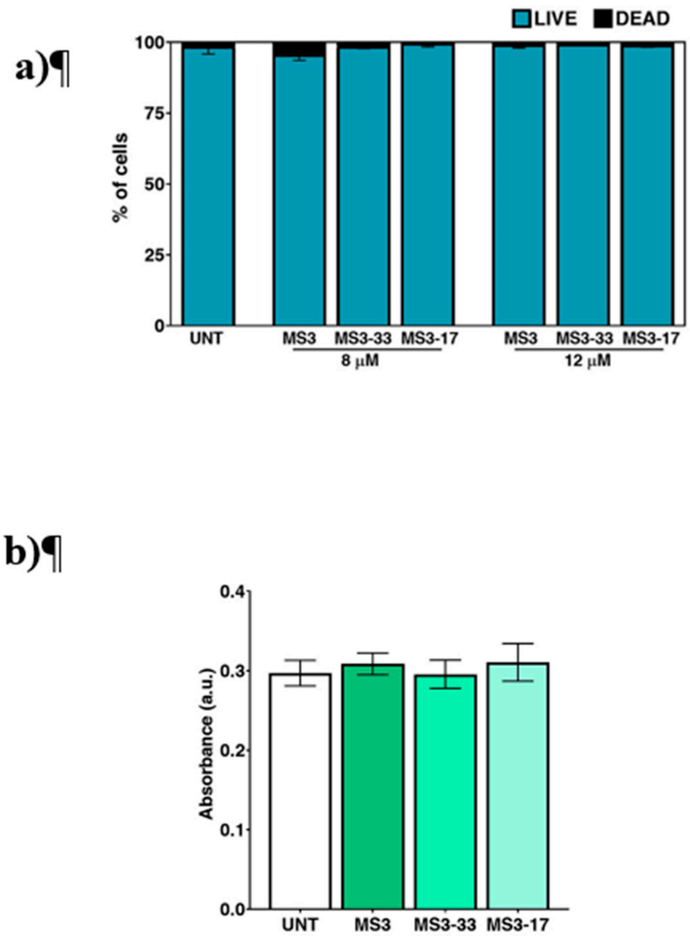

Figure S10. Evaluation of cell viability and metabolic activity of SH-SY5Y cells after treatment with MS3 aptamer and its analogues in comparison with untreated cells (UNT). (**a**) SH-SY5Y cells were incubated for 24 h with the various aptamers at different concentrations as indicated. Percentage of viable and dead cells after trypan blue staining is shown. (**b**) MTT assay on SH-SY5Y cells after 24 h incubation with aptamers (12 μM). For both assays, the values represent the mean of biological

triplicate of two independent experiments; error bars, mean ± SD. No statistical significance, Student's *t*-test.

Table S2. P values of ANOVA statistical analysis followed by Bonferroni multiple comparison test obtained comparing MS3 and its analogues at the indicated concentrations.

APTAMERS	CONCENTRATION			
	**1 μM**	**4 μM**	**8 μM**	**12 μM**
MS3 vs MS3-33	<0,001	<0,001	<0,001	<0,001
MS3 vs MS3-17	0,011	<0,001	<0,001	<0,001

References

1. Karsisiotis, A.I.; Hessari, N.M.A.; Novellino, E.; Spada, G.P.; Randazzo, A.; Webba Da Silva, M. Topological characterization of nucleic acid G-quadruplexes by UV absorption and circular dichroism. *Angew. Chemie - Int. Ed.* **2011**, *50*, 10645–10648, doi:10.1002/anie.201105193.
2. del Villar-Guerra, R.; Trent, J.O.; Chaires, J.B. G-Quadruplex Secondary Structure Obtained from Circular Dichroism Spectroscopy. *Angew. Chemie Int. Ed.* **2018**, *57*, 7171–7175, doi:10.1002/anie.201709184.
